# Prediction of polyreactive and nonspecific single-chain fragment variables through structural biochemical features and protein language-based descriptors

**DOI:** 10.1186/s12859-022-05010-4

**Published:** 2022-12-05

**Authors:** Hocheol Lim, Kyoung Tai No

**Affiliations:** 1 The Interdisciplinary Graduate Program in Integrative Biotechnology and Translational Medicine, Yonsei University, Incheon, 21983 Republic of Korea; 2 Bioinformatics and Molecular Design Research Center (BMDRC), Incheon, 21983 Republic of Korea; 3 Baobab AiBIO Co., Ltd., Incheon, 21983 Republic of Korea

**Keywords:** Antibody design, Nonspecificity, Polyreactivity, Single-chain fragment variable, Machine Learning, Artificial Intelligence

## Abstract

**Background:**

Monoclonal antibodies (mAbs) have been used as therapeutic agents, which must overcome many developability issues after the discovery from in vitro display libraries. Especially, polyreactive mAbs can strongly bind to a specific target and weakly bind to off-target proteins, which leads to poor antibody pharmacokinetics in clinical development. Although early assessment of polyreactive mAbs is important in the early discovery stage, experimental assessments are usually time-consuming and expensive. Therefore, computational approaches for predicting the polyreactivity of single-chain fragment variables (scFvs) in the early discovery stage would be promising for reducing experimental efforts.

**Results:**

Here, we made prediction models for the polyreactivity of scFvs with the known polyreactive antibody features and natural language model descriptors. We predicted 19,426 protein structures of scFvs with trRosetta to calculate the polyreactive antibody features and investigated the classifying performance of each factor for polyreactivity. In the known polyreactive features, the net charge of the CDR2 loop, the tryptophan and glycine residues in CDR-H3, and the lengths of the CDR1 and CDR2 loops, importantly contributed to the performance of the models. Additionally, the hydrodynamic features, such as partial specific volume, gyration radius, and isoelectric points of CDR loops and scFvs, were newly added to improve model performance. Finally, we made the prediction model with a robust performance ($\mathrm{AUC}=0.840$) with an ensemble learning of the top 3 best models.

**Conclusion:**

The prediction models for polyreactivity would help assess polyreactive scFvs in the early discovery stage and our approaches would be promising to develop machine learning models with quantitative data from high throughput assays for antibody screening.

**Supplementary Information:**

The online version contains supplementary material available at 10.1186/s12859-022-05010-4.

## Introduction

Monoclonal antibodies (mAbs) have been important biological research tools and therapeutic agents due to their attractive properties, such as specific binding, conformational stability, safety for a human, and manufacturability [1, 2]. One of the most important properties of mAbs is specificity and binding affinity through complementary-determining regions (CDRs) to a specific antigen unique to its target. To discover novel mAbs, animal immunization has been traditionally used and it has limited control over specificity and binding affinity because of the difficulty in controlling antigen presentation to the animal immune system [2]. Advanced in vitro technologies, such as phage and yeast surface display, have enabled the rapid isolation of mAbs and improved control over antigen presentation [2]. However, the antibodies initially identified via either immunization or these display methods are not suitable for therapeutic use and usually have some unfavorable biophysical characteristics such as stability, solubility, viscosity, polyreactivity, and so on [1].

Therapeutic mAbs must have the desired biophysical properties. The use of mAbs as therapeutics needs to optimize several important properties, such as binding affinity, specificity, folding stability, solubility, pharmacokinetics, effector functions, and other compatibilities with additional antibody or cytotoxic drugs [2]. Although each property can be addressed through screening large antibody libraries, it is difficult to simultaneously optimize multiple properties of mAbs with the screening methods. Attempts to divide and conquer the properties sequentially are limited by the fact that optimizing one property can worsen other properties. The computational antibody-design methods have been developed to overcome the complexity of optimizing multiple properties of mAbs [2].

Many computational tools have been developed to predict the developability of mAbs at an early stage, which includes high aggregation, and poor stability. The predictive tools for aggregation risk have been developed through the identification of chemical modifications in CDRs [3, 4], semi-empirical methods based on spatial-aggregation-propensity [5–7], and machine learning methods predicting the hydrophobic chromatography retention time [8, 9] and the physicochemical properties such as viscosity and isoelectric point [10]. Many computational tools for predicting stability-enhancing mutations have been developed through the phylogenetic information from multiple sequence alignments, the biomolecular simulations for thermodynamic energies [11–13], CDR-dependent position-specific-scoring-matrix [14], and other machine learning methods [15–17] trained with large databases such as ProTherm [18, 19] and TS50 [17]. Furthermore, the developability scores of mAbs have been quantified to help eliminate undesirable mAbs at an early stage through the Developability Index [5] and Therapeutic Antibody Profiler [20], which integrated the aggregation propensity, CDR lengths, and distributions of hydrophobicity and charges on a surface.

Although current antibody discovery methods focused on the generation of mAbs or fragments with high specificity on target, some mAbs can strongly bind to one target and weakly bind to additional antigens. Polyreactivity is called nonspecificity and is an important property because polyreactive mAbs can show reactivity for diverse off-target epitopes. Early assessment of polyreactivity is important in clinical development, which can allow for the prevention of potentially poor candidates. Because polyreactivity of mAbs is linked to poor antibody pharmacokinetics [21], the experimental assessments of polyreactivity have been performed with an enzyme-linked immunosorbent assay (ELISA) [22], protein biochip-based ELISAs [23], and Fluorescence-Activated Cell Sorting (FACS)-based high-throughput selections [24–26]. Because the experimental assessments are usually time-consuming, expensive, and tedious, the computational prediction for polyreactivity help assess mAbs and narrow the search space early. The computational analysis tools for polyreactivity have been developed by identifying the biochemical features in CDRs of polyreactive mAbs, which showed the enrichment of glycine, tryptophan, valine, and arginine motifs [25], an increase in inter-loop crosstalk [27], neutral binding surface [27], high isoelectric points [28], constrained β-sheet structures [29], longer CDRH/L3 loops [30], and the occurrence of glutamine residues [30]. Recently, Harvey et al. showed machine learning (ML) models to assess the polyreactivity of nanobodies from protein sequences [26]. The ML models would help the antibody design and diminish experimental efforts because the ML models can allow the quantifications of the polyreactivity of mAbs.

Machine learning (ML) in protein engineering learns the information from data to predict the protein properties of new variants. Prediction models with ML can accelerate the optimization of protein properties by evaluating the new variants, separating the grain from the chaff, and diminishing experimental efforts. To build the ML models, suitable protein descriptors are required to obtain information in protein sequences. For example, one-hot-encoding of amino acids and single amino acid properties can be used to describe protein sequence as a bottom-up approach [31]. However, obtaining meaningful labels and annotations from the explosively growing protein sequences databases needs expensive and tedious experimental resources [32]. Advanced natural language processing (NLP) techniques are applied to self-supervised learning with unlabeled protein sequences in large protein databases, which may extract evolutionary information from protein sequences [32–35]. In addition to protein sequences, extracting useful information from protein structures is important because a protein function is directly related to and depends on its unique 3D protein structure. Figuring protein structures out is known as the folding problem and the prediction of protein structures from protein sequences has been a long-lasting challenge [36]. In the biennial Critical Assessment of protein Structure Prediction conference, deep learning methods such as AlphaFold and trRosetta outperformed other traditional methods [37, 38], and the more advanced methods such as AlphaFold2 and RoseTTA fold showed a better performance with three-track network architectures [36, 39].

In this work, we made prediction models for the polyreactivity of single-chain fragment variables (scFv) with antibody features and NLP descriptors. First, we calculated sequence- and structure-based antibody features of scFvs. In the sequence-based features, we calculated net charges and lengths of CDR loops. To obtain structure-based features, we predicted protein structures of scFvs with trRosetta. And then we calculated aggregation scores, solvent-accessible surface area, and hydrodynamic properties of scFvs. We investigated the classifying performances of the antibody features for polyreactivity with the area under the curve (AUC) and p-values. Second, we made 20 prediction models for the polyreactivity with the antibody features (F46) and NLP descriptors (UniRep, TAPE, ESM-1b, and ESM-1v) using four machine-learning algorithms (GBM, LGBM, RF, and XGB). Third, we made 16 prediction models with the concatenated descriptors of the antibody features and NLP descriptors to improve model performance. Fourth, we made 14 ensemble models with average- and linear regression-based methods using the 36 prediction models. The prediction models for polyreactivity would help detect the polyreactive scFvs in an early stage and our approaches would help develop machine learning models with high throughput data for antibody screening.

## Methods

### Dataset construction

The polyreactivity dataset of single-chain fragment variables was derived from the ProtaBank [40] and the high-throughput nonspecificity assays by Kelly et al. [25], where a FACS was used to sort depending on whether the scFvs bind to either the soluble membrane preparations or soluble cytosolic preparations in HEK or Sf9 cells, or not. We performed dataset preparation with three steps. First, we removed the duplicate sequences with the same sequences and identical annotations (nonspecific or not). Second, we removed the ambiguous sequences with the same sequences and different annotations. Third, we added pre-gene and post-gene overhang sequences to make full sequences of the scFvs. Finally, We obtained 19,426 sequences, containing the 8867 polyreactive scFvs and 10,559 non-polyreactive scFvs. For the supervised classification task, we prepared a stratified split with an 80% training set (15,540) and a 20% test set (3886) in Python using the Scikit-learn package with a fixed random seed [41].

### Performance metrics and statistical analysis

Performances of the prediction models were evaluated using the area under the receiver operating characteristics curve (AUC), accuracy, precision, recall, and F1-score metrics. The AUC in the ROC curve is a performance measurement for classification problems at various threshold settings and indicates how much the prediction model can distinguish the polyreactivity of scFvs. The accuracy is the ratio of the correctly predicted polyreactive and non-polyreactive scFvs to all the experimental polyreactive and non-polyreactive scFvs in the given data set, which represents how the model can correctly classify the polyreactive and non-polyreactive scFvs out of the data set. The precision score is the ratio of correctly predicted polyreactive scFvs to the total predicted polyreactive scFvs in the given data set, which represents the ability to identify all polyreactive scFvs without any non-polyreactive scFvs. The recall score is the ratio of correctly predicted polyreactive scFvs to all the experimental polyreactive scFvs in the given data set, which represents the ability to correctly predict the polyreactive scFvs out of the experimental polyreactive scFvs. F1-score is the harmonic mean of precision and recall scores, which is an alternative to accuracy. In accuracy, precision, recall, and F1-score metrics, we used the criteria of 0.5.

Statistical difference between two groups of polyreactive and non-polyreactive mAbs in each factor was analyzed by the Student’s *t*-test and two-tailed tests. The p value was used to indicate a statistically significant difference, where *p value < 0.05, **p value < 0.01, and ***p value < 0.001 are considered in this work.

### Homology modeling and protein structure preparation

Homology modeling was performed with transform-restrained Rosetta (trRosetta) [38]. The trRosetta is a deep residual-convolutional network from multiple sequence alignments to make information on the relative distances and orientation of all residue pairs in the protein [38]. And then the restrained minimization was performed to make a protein structure with a fast Rosetta model building protocol with the information from the network [38].

All protein structures from homology modeling were prepared in the following steps. All hydrogen atoms in the protein structures were removed and re-added to the protein structures at pH 7.0. Their positions were optimized with the PROPKA3 implemented in the Maestro program [42]. And then the restrained energy minimization was performed on all protein structures with OPLS3 in the Maestro program within 0.3 Å root mean square deviation [43].

2–4. Aggregation propensities and solvent-accessible surface area.

AggScore [7] is the prediction model for protein aggregation, which is one of the most routinely encountered developability issues [44]. Because the AggScore uses the distribution of hydrophobic and electrostatic patches on the surface of the 3D protein structures and uses the intensity and relative orientation of the surface patches [7], the application domain includes the antibody. Zyaggregator predicts the effects of mutations on the protein aggregation propensity with the physicochemical properties of amino acids [45]. The Zyaggregator score is the sum of Zyaggregator profile Z-scores, whereas the Zyaggregator_p is the normalized score for comparing the proteins which have different lengths. Solvent-accessible surface area (SASA) is the surface of a protein that solvent molecules (water molecules) can access and a probe with the van der Waals radius of a solvent molecule sweeps by rolling over a protein. We calculated the SASA of all hydrophobic atoms (All Hydrophobic SASA) and the exposed hydrophobic atoms (Exposed Hydrophobic SASA). The AggScore, Zyaggregator, and SASA were calculated with the command-line script ‘calc_protein_descriptors.py’, implemented in Schrodinger suite ver. 2018–3.

### Hydrodynamic properties

Hydrodynamic properties of scFvs were calculated with HullRad [46], which uses a convex hull to calculate the smallest convex envelopes with a set of points and to model a hydrodynamic volume of a protein [46]. The 13 factors for hydrodynamic properties are partial specific volume (${\text{v}}_{{{\text{bar}}}}$, ${\text{mL}}/{\text{g}}$), anhydrous volume sphere radius (${\text{R}}_{{\text{o}}}$, Å), the anhydrous radius of gyration (${\text{R}}_{{\text{g}}}$, Å), maximum dimension (${\text{D}}_{{{\text{max}}}}$, Å), axial ratio, frictional ratio (${\text{f}}/{\text{f}}_{0}$), translational diffusion coefficients (${\text{D}}_{{\text{t}}}$, ${\text{cm}}^{2} /s$), translational hydrodynamic radius (${\text{R}}_{{{\text{trans}}}}$, Å), sedimentation coefficients (${\text{s}}$, ${\text{sec}}$), rotational diffusion coefficients (${\text{D}}_{{\text{r}}}$, ${\text{s}}^{ - 1}$), rotational hydrodynamic radius (${\text{R}}_{{{\text{rot}}}}$, Å), tumbling correlation time (${\text{tauC}}$, ${\text{ns}}$), and asphericity. The detailed mathematical equations of the factors are well described in Flemin et al. [46].

### The lengths and G/Q/R/V/W motifs of CDR loops

Delimitation and numbering of CDR regions in all scFv antibodies were performed with AbRSA [47], where the 40% similarity and Chothia scheme [48] were used. In the CDR lengths, the lengths of the whole CDR regions and only CDR3 regions were calculated with the concatenated sequences of all CDR regions and only CDR3 regions. In the CDR3-G/Q/R/V/W motifs, the occurrences of the glycine, glutamine, arginine, valine, and tryptophan residues in CDR3 regions were calculated with the counts and count-to-length ratio using the concatenated CDR3 regions.

### Isoelectric points (IEP)

Isoelectric points (IEP) are the pH, where a molecule has no net electric charge or the statistical mean of the electricity of a molecule is neutral. To estimate the effects of CDR on IEP, we subdivided IEPs into three classes (whole-IEP, CDR-IEP, and CDR3-IEP). The IEP values were predicted with the DTASelect algorithm [49] implemented in pIR [50]. To calculate whole-IEP, CDR-IEP, and CDR3-IEP, the linear approximations were performed with the 25 experimental antibodies’ IEPs [51] and 41,943 experimental peptides’ IEPs [52] through Eqs. (1) and (2).

1 $$IEP_{antibody} = 2.0306{*}IEP_{DTASelect} - 7.8541$$

2 $$IEP_{peptide} = 1.1552*IEP_{DTASelect} - 0.8839$$

We applied $IEP_{antibody}$ to the calculation of whole-IEP and $IEP_{peptide}$ to the calculations of CDR-IEP and CDR3-IEP. The concatenated sequences were used to calculate the CDR-IEP and CDR3-IEP.

### Nanobody polyreactivity

The two prediction models for the polyreactivity of nanobodies were developed by Harvey et al. [26], which are one-hot embedding (OneHot-CDRS) and 3-mer embedding (3MER-CDRS) logistic regression models. The OneHot-CDRS model learned weights for each amino acid type at each position in the CDR sequences, whereas the 3MER-CDRS model learned weights for each motif of polyreactive nanobodies [26]. Although the models were applied to 19,426 scFvs, the polyreactivity scores of 16,337 scFvs were predicted. The AUC scores of OneHot-CDRS and 3MER-CDRS models were calculated with only 16,337 scFvs.

### Protein language-based descriptors (UniRep, TAPE, ESM-1b, and ESM-1v)

Natural language processing (NLP) techniques have been applied to extracting useful evolutionary information from unlabeled protein sequences with self-supervised learning [32–35]. We used UniRep, TAPE, ESM-1b, and ESM-1v descriptors for the NLP-based protein sequence descriptors. The UniRep used the UniRef50 database and was based on a four-layer multiplicative LSTM with 256 hidden units, leading to 18.2 M parameters and 1900 features [33]. The Tasks Assessing Protein Embeddings (TAPE) used the Pfam database and was based on a 12-layer Transformer with a hidden size of 512 units and 8 attention heads, leading to 38 M parameters and 768 features [32]. The Evolutionary Scale Modeling-1b (ESM-1b) used the high-diversity sparse UniRef50 dataset and was based on a 33-layer Transformer with a hidden size of 5120 units and 20 attention heads, leading to 650 M parameters and 1280 features [34]. The Evolutionary Scale Modeling-1v (ESM-1v) had the same architecture as ESM-1b (650 M parameters and 1280 features), but ESM-1v are ensembles of 5 models, used the UniRef90 dataset, and employed zero-shot inference to predict a new class unseen in training sets [35]. The UniRep descriptors were constructed with a concatenation of average hidden unit outputs, the final hidden unit, and the final cell, whereas the TAPE, ESM-1b, and ESM-1v descriptors were constructed with average hidden unit outputs.

#### Machine learning algorithms, hyperparameter tuning, and ensemble learning

Prediction models for polyreactivity of single-chain antibody fragments were constructed using the gradient boosting (GBM) classifier, the random forest (RF) classifier, the light GBM (LGBM) classifier, and the extreme gradient boosting (XGB) classifier models. The GBM combines many weak-leaning models, such as a decision tree, to make a strong prediction model and is based on additive expansions in a forward stage-wise fashion [41, 53]. The RF is a meta-classifier with many classifying decision trees on various subsamples of the dataset, and it uses averaging to improve the predictive accuracy and to control overfitting problems [41]. The LGBM and XGB use the gradient boosting framework, but the difference is how to grow decision trees. The LGBM builds each decision tree in a leaf-wise fashion [54], whereas the XGB builds each decision tree in a depth-wise fashion [55].

In training, we used tenfold cross-validation with GridSearchCV in the Scikit-learn package [41] using hyperparameter settings (Additional file 1: Table S1). The best hyperparameters from the GridSearchCV were selected with the performance in cross-validation sets. And then the final model with the best hyperparameters was re-trained with all training sets. Some hyperparameters in GBM were incorporated for tuning the models; the number of boosting stages (n_estimators), the maximum depth of the individual regression estimators (max_depth), the number of features to consider when finding the best split (max_features), and the boosting learning rate (learning_rate) [41, 53]. Some hyperparameters in LGBM were incorporated for tuning the models; n_estimators and learning_rate [54]. Some hyperparameters in RF were incorporated for tuning the models; n_estimators and max_features [41]. Some hyperparameters in XGB were incorporated for tuning the models; n_estimators, max_depth, and learning_rate [55].

#### Ensemble learning through a meta-learning classifier

Ensemble learning through a meta-learning classifier was performed with average-based (AVG) and linear regression-based (LR) methods. In the AVG method, we calculated the average of the probabilities from the pre-trained selected models. In the LR method, we trained a simple linear regression model without an intercept term using the probabilities from the pre-trained selected models in the training set. The difference between the AVG and LR strategies is that the contribution to the final probability of each pre-trained model is the same in the AVG strategy, whereas the contribution of each pre-trained model is not the same in the LR strategy. Machine learning learns how to best use input features and information to predict nonspecificity, whereas ensemble learning learns how to best use the machine learning models to predict nonspecificity.

## Results

**Fig. 1:**
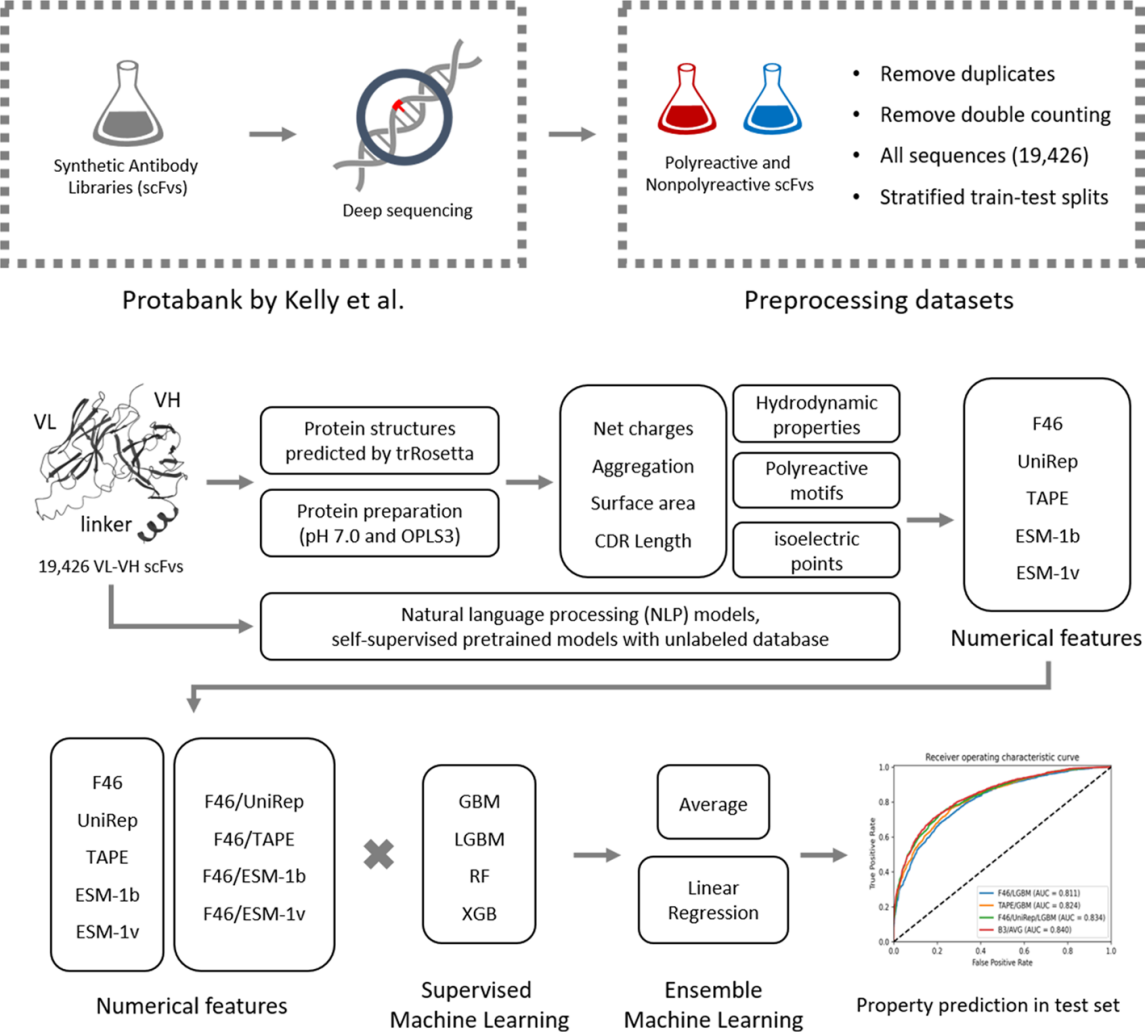
Workflow to make prediction models for polyreactivity in this work

Computational prediction of the polyreactivity of antibodies is important in evaluating the developability of antibodies at an early stage. The workflow to make computational prediction models for the polyreactivity in scFvs is shown in Fig. 1.

## Each performance of biochemical patterns in scFvs for polyreactivity

Most prediction methods for polyreactivity have focused on the identification of the biochemical features which the polyreactive antibodies [25–30]. The biochemical patterns in polyreactive antibodies have been analyzed through an increase in neutral binding surface [27], longer CDRH/L3 loops, an increase of glycine, tryptophan, valine, and arginine motifs [25], the occurrence of glutamine residues [30], and high isoelectric points [28]. To investigate each classifying performance of the sequence-based and structure-based features, we predicted 19,426 scFv antibody structures with trRosetta and calculated the area under the receiver operating characteristic curves (AUC) with the 51 biochemical features (Additional file 1: Table S3 and Additional file 1: Table S4). The distributions of the statistically significant features (p value < 0.001) are illustrated in Additional file 1: Figure S1 and Additional file 1: Figure S2.

A slightly hydrophilic and neutral-charged binding surface can have weak interactions with various ligands [27]. To investigate the classifying performance of the increased neutral binding surfaces, we calculated the area under the ROC curves (AUC) with the net charges of CDR loops, spatial aggregation propensities (SAP), and solvent-accessible surface area (SASA) with the predicted structures. Firstly, we delimited the CDR of scFvs with the Chothia scheme and calculated the net charges of all CDRs, CDR1, CDR2, and CDR3 loops. The AUC scores of the net charges of all CDRs and CDR1, CDR2, and CDR3 loops are 0.521, 0.556, 0.413, and 0.521, respectively. Secondly, we predicted the SAP of scFvs with AggScore and Zyggregator with the predicted structures. The AUC scores of AggScore, Zyggregator, and Zyggregator_p are 0.506, 0.489, and 0.541, respectively. Thirdly, we calculated the SASA of all hydrophobic atoms and the exposed hydrophobic atoms with the predicted protein structures. The AUC scores of the hydrophobic SASA of exposed residues and all residues are 0.512 and 0.502.

In addition to the SAP and SASA, the molecular-scale hydrodynamic effects are related to the cavity-ligand binding due to the capillary fluctuations [56]. Hydrodynamic properties can be used to estimate the size and shape of the proteins in solution [46] because the hydrodynamic radius in a protein involves the motion of the protein relative to the aqueous solvent where the protein is dissolved [57]. We calculated the 14 hydrodynamic properties and measured the classifying performance for polyreactivity in scFvs. In the 14 properties, the AUC scores of the anhydrous radius of gyration, asphericity, and frictional ratio are 0.628, 0.621, and 0.600, respectively. The three factors of the gyration radius, frictional ratio, and asphericity are related to the hydration effect [46, 58], they are relatively better predictive of the polyreactivity in scFvs than other hydrodynamic properties.

Lecerf et al. reported that the hydrophobicity and propensity for aggregation of mAbs are associated with the longer CDRH/L3 loops, but there is no significant correlation between the size of hypervariable loops and the polyreactivity [30]. To investigate the correlation between the length of CDR loops and polyreactivity, we measured the lengths of all CDRs and CDR-1/2/3 loops in scFvs. The AUC scores of the lengths of all CDRs, CDR1, CDR2, and CDR3 are 0.482, 0.393, 0.530, and 0.545. The lower AUC scores mean that longer CDR lengths cannot distinguish the nonspecificity in scFvs. On the other hand, shorter lengths of CDR1 showed a relatively better classifying performance ($\mathrm{AUC}=0.607$). The results were in agreement with Lecerf et al. [30], where the mAbs with shorter CDR loops might tend to reduce the risk of polyreactivity.

Enrichment of the glycine-, glutamine-, arginine-, valine-, and tryptophan motifs in CDR-H3 is associated with polyreactivity [25, 30]. To investigate the classifying performance of each motif, we calculated the AUC scores of the number and ratio of each motif in CDR3 and CDR-H3 (G, Q, R, V, W, VV, and WW motifs). Most motifs showed low AUC scores between 0.450 and 0.550, but the Trp motifs even misled the classification ($\mathrm{AUC}=0.383$).

Isoelectric points (IEP) of mAbs are important in solution behavior and related to viscosity [10]. Because therapeutic antibodies need to be positively charged for efficient fluid-phase endocytosis at the physiological pH of 7.4, an IEP in the range of 8–9 is desirable. To investigate the correlation between IEPs and polyreactivity, we predicted the IEPs of scFvs based on the full sequences and concatenated CDR loops. Because most IEP prediction methods have been developed for peptides and proteins [49, 52], we corrected the predicted IEP with experimental IEPs from peptides and antibodies and prediction models for peptides and antibodies (Additional file 1: Figure S3). To predict the IEPs for CDR loops, we collected the experimental IEPs of 41,943 peptides and made a prediction model of ${\mathrm{R}}^{2}=0.9845$ and $\mathrm{RMSE}=0.2743$. Whereas, to predict the IEPs for antibodies, we collected the experimental IEPs of 25 antibodies and made a prediction model of ${\mathrm{R}}^{2}=0.9603$ and $\mathrm{RMSE}=0.1669$. The AUC scores of the predicted IEPs of CDRs and scFvs are 0.504 and 0.535, respectively.

Although the length of CDR1, anhydrous gyration radius, frictional ratio, and asphericity showed relatively high AUC over 0.6, the 40 biochemical features in scFvs showed low AUC if each pattern was used to classify the polyreactive scFvs alone. Because a single biochemical feature in scFvs is not enough to distinguish and predict the polyreactive scFvs, it is necessary to build machine learning models to predict the polyreactivity of the scFvs.

## Machine learning models with biochemical features

**Fig. 2:**
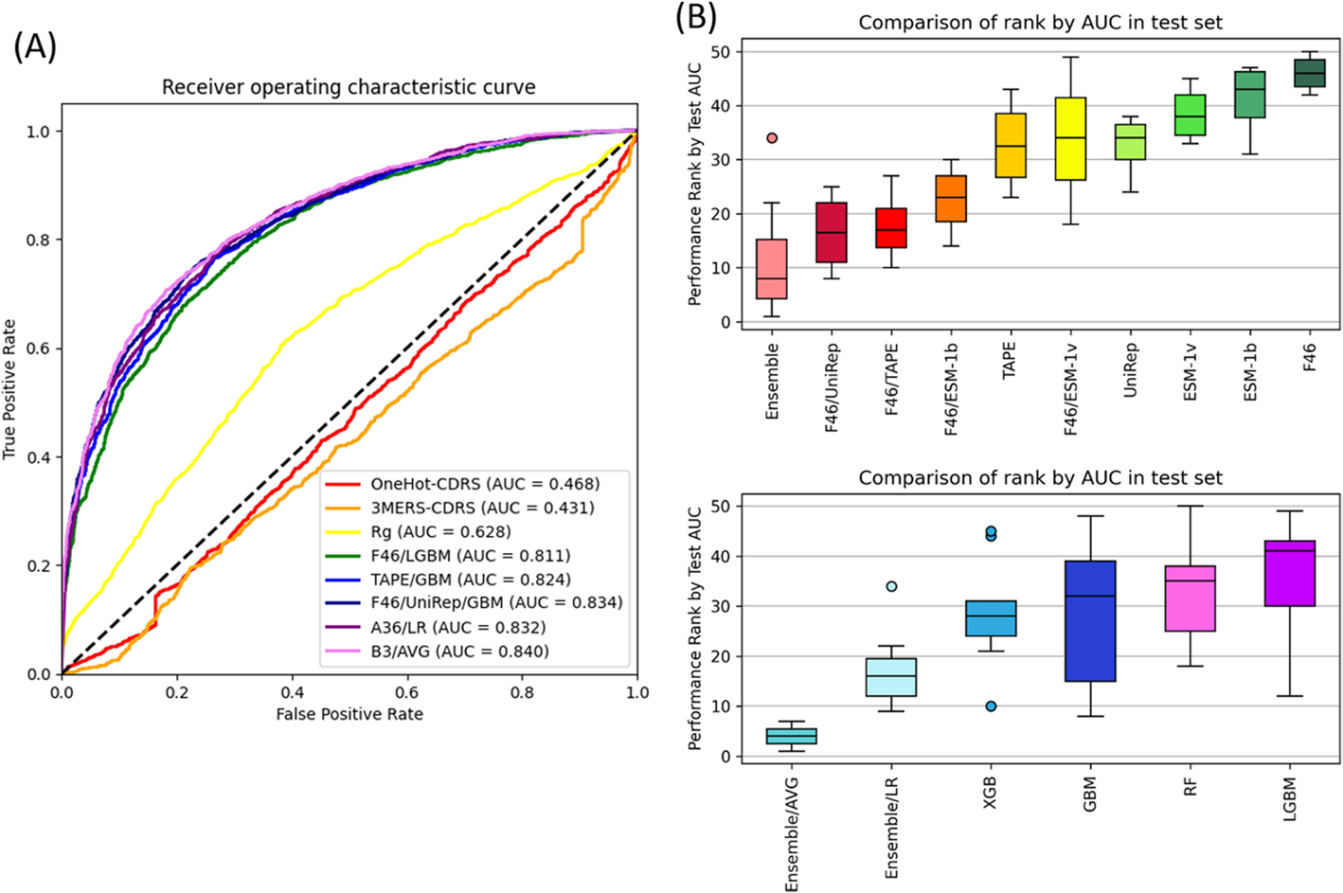
Performance metrics of the models in this work. **A** ROC plots for the best models from the combinations of descriptors and algorithms. The ‘OneHot-CDRS’ and ‘3MERS-CDRS’ are the reported models by Harvey et al. (ref. 26) for nanobody and we used them as baselines. The ‘Rg’ is the anhydrous radius of gyration and the best single factor in this work. The best models were trained with the optimal hyperparameter after grid search. The AUC metric was measured in the test set. **B** Boxplot plot for the relative comparison rank of descriptors and algorithms. We put all the models together and ranked the descriptors and algorithms by the AUC metric in the test set

Machine learning models can utilize the information of the scFvs to predict the polyreactivity of the scFvs. We developed machine learning models with the combination of the biochemical features and four NLP-based descriptors (UniRep, TAPE, ESM-1b, and ESM-1v). To compare the performance of the models, we used the AUC scores in the test set after tenfold cross-validation and refitting for our-own models. And then we built baselines with the best AUC of the single features and the AUC for the two previously developed models for antibody fragments by Harvey et al. [26] (one-hot-CDRS and 3mer-CDRs). The ROC plots of the two models are illustrated in Fig. 2A.

The anhydrous radius of gyration showed the best performance ($\mathrm{AUC}=0.628$) in the single biochemical features for predicting polyreactive scFvs. To build the baselines with the previously developed models, we applied the two polyreactive prediction models for antibody fragments by Harvey et al. [26] (one-hot-CDRS and 3mer-CDRS) to the scFvs. Due to the IMGT numbering scheme with ANARCI in Harvey et al. [26], the polyreactivities of only 16,337 scFvs were used to calculate the AUC values. The AUC values of one-hot-CDRS and 3mer-CDRS for scFvs are 0.467 and 0.428.

To find the best model with the biochemical features, we made four machine learning models (GBM, LGBM, RF, and XGB) with 46 biochemical patterns (F46) except for two SASA and three aggregation factors. The performance metrics of the four models are summarized in Table 1. The optimal hyperparameters of the models are summarized in Additional file 1: Table S2. The prediction model from F46 and LGBM showed the best performance in the test set prediction ($\mathrm{AUC}=0.811$). The best model was trained with the optimal hyperparameter (learning_rate = 0.01 and n_estimators = 500). Accuracy, precision, recall, and F1-score of the best model (F46/LGBM) are 0.762, 0.732, 0.756, and 0.744 in the training set, whereas those are 0.731, 0.700, 0.720, and 0.710 in the test set. The ROC plot of the best model (F46/LGBM) is illustrated in Fig. 2A.

**Table 1:** Performance of models with different ML algorithms and single descriptors

Descriptor	Method	Train AUC	Valid AUC	Test AUC	Accuracy	Precision	Recall	F1-score
F46	GBM	0.917 ± 0.006	0.705 ± 0.129	0.805	0.730	0.753	0.607	0.672
	LGBM	0.858 ± 0.009	0.704 ± 0.124	0.811	0.731	0.700	0.720	0.710
	RF	1.000 ± 0.000	0.697 ± 0.123	0.795	0.728	0.722	0.658	0.689
	XGB	0.951 ± 0.003	0.701 ± 0.125	0.810	0.732	0.705	0.710	0.708
UniRep	GBM	1.000 ± 0.000	0.591 ± 0.177	0.821	0.747	0.741	0.686	0.712
	LGBM	0.926 ± 0.006	0.606 ± 0.178	0.816	0.734	0.704	0.722	0.713
	RF	1.000 ± 0.000	0.575 ± 0.178	0.815	0.736	0.728	0.671	0.699
	XGB	0.999 ± 0.000	0.596 ± 0.181	**0.824**	0.740	0.718	0.709	0.713
TAPE	GBM	1.000 ± 0.000	0.647 ± 0.160	**0.824**	0.745	0.741	0.679	0.709
	LGBM	0.919 ± 0.005	0.657 ± 0.155	0.810	0.731	0.703	0.713	0.708
	RF	1.000 ± 0.000	0.638 ± 0.160	0.815	0.745	0.747	0.666	0.704
	XGB	0.998 ± 0.000	0.651 ± 0.156	0.822	0.746	0.729	0.706	0.717
ESM-1b	GBM	0.979 ± 0.003	0.603 ± 0.182	0.807	0.727	0.742	0.616	0.673
	LGBM	0.922 ± 0.006	0.608 ± 0.176	0.807	0.730	0.697	0.722	0.710
	RF	1.000 ± 0.000	0.593 ± 0.176	0.814	0.736	0.730	0.669	0.698
	XGB	0.998 ± 0.000	0.604 ± 0.177	0.821	0.741	0.718	0.713	0.716
ESM-1v	GBM	1.000 ± 0.000	0.594 ± 0.150	0.819	0.743	0.761	0.639	0.695
	LGBM	0.919 ± 0.005	0.602 ± 0.172	0.813	0.730	0.694	0.729	0.711
	RF	1.000 ± 0.000	0.582 ± 0.171	0.816	0.740	0.735	0.674	0.703
	XGB	0.949 ± 0.002	0.597 ± 0.175	0.808	0.728	0.702	0.701	0.701

The bold means the best performance, the AUC score in the test set

We calculated the feature importance in the best model to investigate the contributions of the 46 biochemical features to the best model (F46/LGBM). The top 11 important features in the best model had 50.87% contributions to the performance, which are the net charge of the CDR2 loop (7.25%), partial specific volume (6.00%), the isoelectric point of CDR loops (5.90%), the anhydrous radius of gyration (4.45%), the ratio of tryptophan residues in CDR-H3 (4.41%), the isoelectric point of scFv (4.21%), the CDR1 length (4.01%), the anhydrous volume sphere radius (3.97%), the CDR2 length (3.85%), the count of glycine residues in CDR-H3 (3.61%), and sedimentation coefficients (3.21%). The net charge of the CDR2 loop and the counts and ratios of tryptophan and glycine residues are associated with the known biochemical features of the neutral binding surface [27] and the enrichment of tryptophan and glycine motifs [25]. The four hydrodynamic properties (partial specific volume, the anhydrous radius of gyration, the anhydrous volume sphere radius, and sedimentation coefficients) had 17.63% contributions to the performance of the best model, indicating that the solution behavior is important in the polyreactivity of the scFvs.

## Machine learning models with NLP-based descriptors

Natural language processing methods have been applied to large unlabeled protein sequence data sets through self-supervised learning, which leads to language model-based descriptors. Because the language model-based descriptors can extract evolutionary information from protein sequences, we used four language model-based descriptors (UniRep, TAPE, ESM-1b, and ESM-1v) to extract information from scFvs’ sequences.

To find the best model with the language model-based descriptors, we made 16 machine learning models with the combination of four machine learning algorithms (GBM, LGBM, RF, and XGB) and four language model-based descriptors (UniRep, TAPE, ESM-1b, and ESM-1v). The performance metrics of the 16 models are summarized in Table 1. The ROC plot of the best model (TAPE/GBM) is illustrated in Fig. 2A.

In the UniRep, the prediction model from XGB showed the best performance in the test set ($\mathrm{AUC}=0.824$). The best model (UniRep/XGB) was trained with the optimal hyperparameter (learning_rate = 0.01, max_depth = 10, and n_estimators = 500). The accuracy, precision, recall, and F1-score of the best model (UniRep/XGB) are 0.740, 0.718, 0.709, and 0.713 in the test set, respectively. In the TAPE, the prediction model from GBM showed the best performance in the test set ($\mathrm{AUC}=0.824$). The best model (TAPE/GBM) was trained with the optimal hyperparameter (learning_rate = 0.01, max_depth = 10, max_features = ‘sqrt’, and n_estimators = 1000). The accuracy, precision, recall, and F1-score of the best model (TAPE/GBM) are 0.745, 0.741, 0.679, and 0.709 in the test set, respectively. In the ESM-1b, the prediction model from XGB showed the best performance in the test set ($\mathrm{AUC}=0.821$). The best model (ESM-1b/XGB) was trained with the optimal hyperparameter (learning_rate = 0.01, max_depth = 10, and n_estimators = 500). The accuracy, precision, recall, and F1-score of the best model (ESM-1b/XGB) are 0.741, 0.718, 0.713, and 0.716 in the test set, respectively. In the ESM-1v, the prediction model from GBM showed the best performance in the test set ($\mathrm{AUC}=0.819$). The best model (ESM-1v/GBM) was trained with the optimal hyperparameter (learning_rate = 0.05, max_depth = 15, max_features = ‘log2’, and n_estimators = 3000). The accuracy, precision, recall, and F1-score of the best model (ESM-1v/GBM) are 0.743, 0.761, 0.639, and 0.695 in the test set, respectively.

To compare the performance of the machine learning models from biochemical features and language model-based descriptors, we used the AUC in tenfold cross-validation test sets and five metrics in the test set (AUC, accuracy, precision, recall, and F1-score metrics).

In the AUC metric in tenfold cross-validation sets, the F46/LGBM model showed the best performance ($0.704\pm 0.124$), whereas the other models had relatively low mean and high standard deviations of AUC in tenfold cross-validation sets. It indicated that the language model-based descriptors are more sensitive to the data splits than the 46 biochemical features. In the AUC metric in the test set, the TAPE/GBM model showed the best performance (0.824), whereas the other models also showed a robust performance over 0.8. The ROC plot of the best model (TAPE/GBM) is illustrated in Fig. 2A. In the accuracy in the test set, the TAPE/GBM model showed the best accuracy (0.745), whereas the other models also had similar accuracy over 0.730. In the precision metric in the test set, the ESM-1v/GBM showed the best precision score (0.761), whereas the other models also had a robust precision score of over 0.7. In the recall metric in the test set, the F46/LGBM model showed the best recall score (0.720), whereas the TAPE/GBM and ESM-1v/GBM models showed relatively low recall scores (0.679 and 0.639, respectively). In the F1-score metric in the test set, the ESM-1b/XGB showed the best F1-score (0.716), whereas the ESM-1v/GBM model showed the worst F1-score (0.695) and the other models showed a robust F1-score over 0.7.

## Machine learning models with both biochemical features and language model-based descriptors

To improve model performance, we concatenated the 46 biochemical features and four language model-based descriptors and made the four descriptors (F46/UniRep, F46/TAPE, F46/ESM-1b, and F46/ESM-1v). And then we made the 16 machine learning models with the combinations of the four descriptors and four machine learning algorithms (GBM, LGBM, RF, and XGB), the performance metrics of which are summarized in Table 2. The optimal hyperparameters of the models are summarized in Additional file 1: Table S2.

**Table 2:** Performance of models with different ML algorithms and the concatenated descriptors

Descriptor	Method	Train AUC	Valid AUC	Test AUC	Accuracy	Precision	Recall	F1-score
F46/UniRep	GBM	1.000 ± 0.000	0.624 ± 0.163	**0.834**	0.759	0.751	0.706	0.728
	LGBM	0.928 ± 0.006	0.638 ± 0.166	0.830	0.746	0.719	0.729	0.724
	RF	1.000 ± 0.000	0.594 ± 0.174	0.823	0.747	0.742	0.683	0.711
	XGB	0.971 ± 0.002	0.624 ± 0.166	0.826	0.752	0.728	0.729	0.729
F46/TAPE	GBM	1.000 ± 0.000	0.669 ± 0.137	0.829	0.748	0.745	0.680	0.711
	LGBM	0.919 ± 0.006	0.676 ± 0.147	0.826	0.746	0.720	0.726	0.723
	RF	1.000 ± 0.000	0.654 ± 0.153	0.823	0.749	0.752	0.671	0.709
	XGB	0.997 ± 0.000	0.668 ± 0.151	0.831	0.748	0.727	0.718	0.722
F46/ESM-1b	GBM	1.000 ± 0.000	0.643 ± 0.154	0.830	0.755	0.746	0.704	0.724
	LGBM	0.882 ± 0.008	0.654 ± 0.157	0.821	0.744	0.709	0.746	0.727
	RF	1.000 ± 0.000	0.622 ± 0.169	0.826	0.750	0.748	0.680	0.713
	XGB	0.954 ± 0.004	0.648 ± 0.153	0.823	0.750	0.721	0.738	0.729
F46/ESM-1v	GBM	0.985 ± 0.002	0.643 ± 0.139	0.814	0.738	0.741	0.654	0.695
	LGBM	0.834 ± 0.010	0.657 ± 0.134	0.802	0.727	0.677	0.766	0.719
	RF	1.000 ± 0.000	0.611 ± 0.165	0.827	0.751	0.750	0.681	0.714
	XGB	0.951 ± 0.003	0.643 ± 0.151	0.822	0.743	0.709	0.742	0.725

The bold means the best performance, the AUC score in the test set

In the F46/UniRep, the prediction model from GBM showed the best performance in the test set ($\mathrm{AUC}=0.834$). The ROC plot of the best model (F46/UniRep/GBM) is illustrated in Fig. 2A. The best model (F46/UniRep/GBM) was trained with the optimal hyperparameter (learning_rate = 0.01, max_depth = 10, max_feature = ‘auto’, and n_estimators = 1000). The accuracy, precision, recall, and F1-score of the best model (F46/UniRep/GBM) are 0.759, 0.751, 0.706, and 0.728 in the test set, respectively. In the F46/TAPE, the prediction model from XGB showed the best performance in the test set ($\mathrm{AUC}=0.831$). The best model (F46/TAPE/XGB) was trained with the optimal hyperparameter (learning_rate = 0.01, max_depth = 10, and n_estimators = 500). The accuracy, precision, recall, and F1-score of the best model (F46/TAPE/XGB) are 0.748, 0.727, 0.718, and 0.722 in the test set, respectively. In the F46/ESM-1b, the prediction model from GBM showed the best performance in the test set ($\mathrm{AUC}=0.830$). The best model (F46/ESM-1b/GBM) was trained with the optimal hyperparameter (learning_rate = 0.01, max_depth = 10, max_feature = ‘auto’, and n_estimators = 500). The accuracy, precision, recall, and F1-score of the best model (F46/ESM-1b/GBM) are 0.755, 0.746, 0.704, and 0.724 in the test set, respectively. In the F46/ESM-1v, the prediction model from RF showed the best performance in the test set ($\mathrm{AUC}=0.827$). The best model (F46/ESM-1v/RF) was trained with the optimal hyperparameter (max_feature = ‘auto’, and n_estimators = 500). The accuracy, precision, recall, and F1-score of the best model (F46/ESM-1b/GBM) are 0.751, 0.750, 0.681, and 0.714 in the test set, respectively.

## Ensemble models to improve the performance

To improve model performance, we performed ensemble learning with the 36 machine learning models from the previous hyperparameter optimization steps. We made 14 ensemble models with the two combination methods and two ensemble learnings (average-based and linear regression-based methods). We ranked the 36 models with the AUC metric in the test set. In one combination, we selected all, the top 10, top 5, and top 3 models in all 36 models, which led to A36, A10, A5, and A3 models, respectively. In the other combination, we selected all models, the top 5, and top 3 models in the best models of nine protein descriptor sets, which led to B9, B5, and B3 models, respectively. The performance metrics are summarized in Table 3.

**Table 3:** Performance of ensemble models with the trained model combinations

Name	Method	Test AUC	Accuracy	Precision	Recall	F1-score
A36	Average-based Ensemble (AVG)	0.836	0.758	0.750	0.703	0.726
A10		0.839	0.764	0.757	0.710	0.733
A5		0.839	0.760	0.755	0.701	0.727
A3		0.839	0.764	0.751	0.724	0.737
B9		0.838	0.754	0.751	0.688	0.718
B5		0.839	0.759	0.748	0.711	0.729
**B3**		**0.840**	0.765	0.755	0.717	0.735
A36	Linear Regression-based Ensemble (LR)	0.832	0.754	0.773	0.653	0.708
A10		0.828	0.748	0.745	0.680	0.711
A5		0.828	0.748	0.745	0.680	0.711
A3		0.831	0.759	0.751	0.706	0.728
B9		0.819	0.743	0.761	0.639	0.695
B5		0.830	0.758	0.750	0.705	0.727
B3		0.825	0.757	0.748	0.705	0.726
TAPE/GBM		0.824	0.745	0.741	0.679	0.712
F46/UniRep/GBM		0.834	0.759	0.751	0.706	0.728

The bold means the best performance, the AUC score in the test set

In the average-based ensemble learning (AVG), the prediction model from B3 showed the best performance in the test set ($\mathrm{AUC}=0.840$). The accuracy, precision, recall, and F1-score of the best model (B3/AVG) are 0.765, 0.755, 0.717, and 0.735 in the test set, respectively. The prediction models from A10/AVG, A5/AVG, A3/AVG, and B5/AVG tied for the second performance in the test set ($\mathrm{AUC}=0.839$). The prediction model from A36/AVG also showed a slightly better performance than the base model (F46/UniRep/GBM), but it showed the worst performance in the average-based ensemble models. In the linear regression-based ensemble learning (LR), the prediction model from A36 showed the best performance in the test set ($\mathrm{AUC}=0.832$). The accuracy, precision, recall, and F1-score of the best model (A36/LR) are 0.754, 0.773, 0.653, and 0.708 in the test set, respectively. The models from LR-based ensemble learning showed worse performance than the baseline (F46/UniRep/GBM), which may be from the overfitting problem.

## Performance comparison of the prediction models.

To compare evaluation metrics in protein descriptors and machine learning algorithms, the performance of the 50 final models was ranked according to the mean value of decreasing order for the AUC in the test set in Fig. 2B. The Ensemble won in protein descriptor ranking, followed by F46/UniRep, F46/TAPE, F46/ESM-1b, TAPE, F46/ESM-1v, UniRep, ESM-1v, ESM-1b, and F46. The Ensemble/AVG won in machine learning algorithm ranking, followed by Ensemble/LR, XGB, GBM, RF, and LGBM.

## Discussion

Antibodies have been successful biological drugs with over 100 molecules approved for therapeutic use and hundreds more in clinical development. Improved high-throughput technologies enable to find of very specific antibodies against targets, but some can show polyreactivity with low affinity for multiple epitopes. Because polyreactive antibodies have potential side effects through multiple epitopes, previous studies have identified the biochemical patterns and characteristics of polyreactive antibodies. Here, we constructed machine learning models to predict the polyreactivity of scFvs with antibody features and NLP descriptors. The computational frameworks to predict the polyreactivity of a given scFv would be useful by evaluating the potential fate of a therapeutic antibody and the potential efficacy with natural immune systems in the early discovery stage. Many studies have focused to identify the biochemical polyreactive features in antibodies [25, 27–30], but the single factors have low AUC performance to classify the polyreactive scFvs. Recently, the in silico method has been developed to predict the polyreactivity of antibody fragment [26], but it focused on nanobodies and the application to scFvs showed low AUC performance. Therefore, computational prediction models for polyreactivity in scFvs in this work could be used to support the isolation of the potential polyreactive scFvs in the process of therapeutic antibody screening. Moreover, similar approaches with the protein structural features from protein structure prediction methods and NLP descriptors would be promising and useful to make machine learning models for industrial enzymes and protein drugs.

Advanced natural language processing technologies have enabled us to learn statistical representations and evolutionary information of protein sequences with continuously increased unlabeled protein sequence databases from advanced sequencing technologies. The NLP-based protein descriptors (UniRep, TAPE, ESM-1b, and ESM-1v) can capture the polyreactive features of scFv sequences to classify the polyreactive scFvs. The machine learning models with the NLP-based descriptors showed moderate performance, but they are more sensitive to data splits than structural features. Many language models have been proposed with large-scale databases of protein sequences over the families of related protein sequences [32–34, 59]. Although we used four NLP descriptors in this work, the more advanced NLP descriptors with large-scale databases would improve the model performance. The NLP protein descriptors have the potential for diverse protein engineering tasks [31] because it enables us to compare the protein sequences too diverse to perform multiple sequence alignment analysis. However, there is an inevitable limitation of the sequence-function gap, because protein functions are from the accurately folded protein structures.

Protein structures form the basis of the structure–activity relationship. Although the protein structural features help the analysis of the relationship, the relatively small number of experimentally determined protein structures has set a limit on wide applications of machine learning and deep learning approaches with the determined protein structures. Protein structure prediction methods such as AlphaFold [37, 39], trRosetta [38], and RoseTTAFold [36], have enabled the rapid generation of protein structural features for machine learning approaches. Although we used the trRosetta method to predict the protein structures of scFvs due to the computational cost, AlphaFold 2 and RoseTTAFold have been known to outperform trRosetta to predict antibody structures [39, 60], which can improve model performance with the structural features from the more accurate protein structures. The more accurate and rapid protein structure prediction methods would accelerate the applications of machine learning and deep learning approaches with protein structures.

Not only the development of protein descriptors but also the advance of machine learning algorithms would help make prediction models. The decision tree models such as GBM, LGBM, RF, and XGB used in this work are ensemble methods with weak learners and are computationally efficient models and have been used in various classification tasks [61, 62]. Although we use only the decision-tree-based ensemble models, there are alternative or possibly better machine learning algorithms. Kernel methods, such as support vector machine (SVM), calculate the similarity between inputs and implicitly project the features into a high dimensional space. The SVM has been successfully applied to various classification tasks [63, 64] and worked well when there is a clear margin of separation between two classes. However, the SVM requires high computational cost and is not suitable for large data sets, because the SVM needs to calculate the similarity between input features. Recently, a canonical deep neural network architecture for tabular data (TabNet) was developed and showed better performance than the decision tree models for some supervised learning and semi-supervised learning tasks [65]. Therefore, to find a suitable combination for the training data, it would be helpful to build models with diverse machine learning algorithms and compare the performance their performance in the future.

## Conclusion

Monoclonal antibodies have been essential biological therapeutic agents, which require optimizing many physical properties for clinical development after in vitro library screening. Polyreactivity is one of the most important properties in clinical development because it leads to poor pharmacokinetic properties and potential poor candidates. We made prediction models with the known polyreactive antibody features and NLP descriptors, where we predicted all scFv protein structures with trRosetta to calculate structure-based features. The best model in this work showed a robust performance ($\mathrm{AUC}=0.840$) with 76.5% accuracy and 75.5% precision rates. Therefore, computational prediction for polyreactivity with our models would help detect the polyreactive mAbs and allow for the prevention of potentially poor candidates in the early discovery stage. Furthermore, our approaches would be promising to make machine learning models with quantitative data from high throughput assays for industrial enzyme and antibody screening.

## Supplementary Information

**Additional file 1**. Supplementary figures and tables.

## Data Availability

The datasets used in the present research are available on the Github repository (https://github.com/hclim0213/Pred_nonspecificity_scFvs).
