# Supplementary material for: Prediction of polyreactive and nonspecific single-chain fragment variables through structural biochemical features and protein language-based descriptors
Source: BMC Bioinformatics. 2022 Dec 5;23:520. doi: 10.1186/s12859-022-05010-4 (PMC9720949; doi:10.1186/s12859-022-05010-4)
Supplement: Supplementary file 1 — Additional file 1. Supplementary figures and tables. [file 12859_2022_5010_MOESM1_ESM.pdf]

Supporting Information for

Prediction of polyreactive and nonspecific single-chain fragment variables through structural biochemical features and protein language-based descriptors.

Hocheol Lim[a,b] and Kyoung Tai No[a,b,c,*]

[a] The Interdisciplinary Graduate Program in Integrative Biotechnology and Translational Medicine, Yonsei University, Incheon 21983, Republic of Korea

[b] Bioinformatics and Molecular Design Research Center (BMDRC), Incheon 21983, Republic of Korea

[c] Baobab AiBIO Co., Ltd., Incheon 21983, Republic of Korea

[*] Corresponding author: Kyoung Tai No (ktno@yonsei.ac.kr)

Supporting information for ‘Prediction of polyreactive and nonspecific single-chain fragment variables through structural biochemical features and protein language-based descriptors’ includes Figures S1 – S2 for the violin plots of the statistically significant factors in scFvs and Figure S3 for correlation plots of the experimental IEPs and predicted IEPs in peptides and antibodies. It also includes Tables S1 – S2 for hyperparameter tuning and Tables S3 – S4 for the classifying performance of biochemical features in scFvs against polyreactivity.

In this study, there are many abbreviations as follows. AUC, Area under the receiver operating characteristics curve; AVG, Average-based ensemble learning; CDR, Complementary-determining regions; ELISA, Enzyme-linked immunosorbent assay; ESM, Evolutionary scale modeling; FACS, Fluorescence-activated cell sorting; GBM, Gradient boosting; IEP, Isoelectric points; LGBM, Light gradient boosting; LR, Linear regression-based ensemble learning; mAbs, Monoclonal antibodies; ML, Machine learning; NLP, Natural language processing; RF, Random forest; ROC, Receiver operating characteristics; SAP, Spatial aggregation propensity; SASA, Solvent-accessible surface area; scFvs, Single-chain fragment variables; SVM, Support vector machine; TAPE, Tasks assessing protein embeddings; trRosetta, Transform-restrained Rosetta; XGB, Extreme gradient boosting.

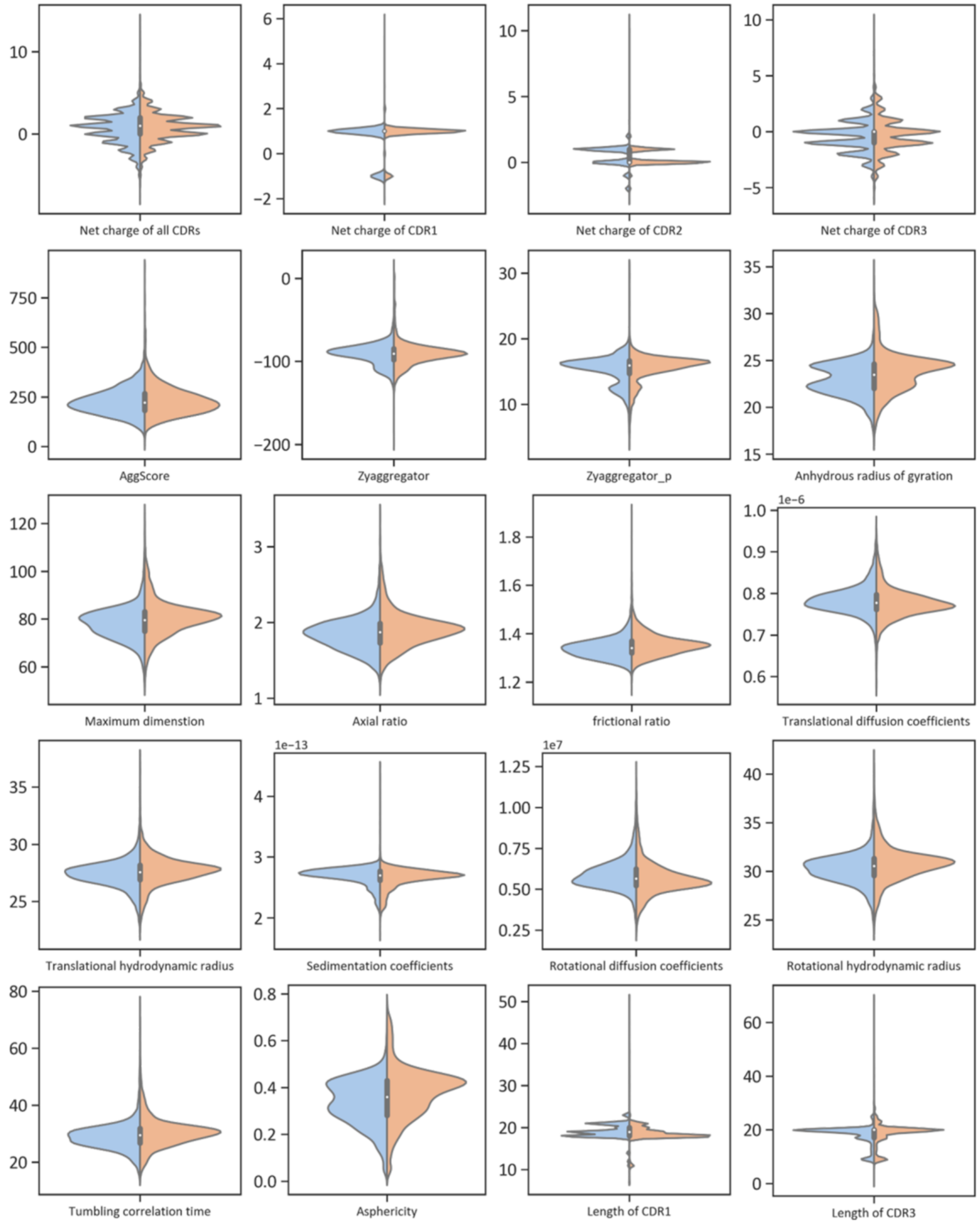

Figure S1. Violin plots for the physicochemical properties of scFvs.

Polyreactive mAbs are colored in red, while nonpolyreactive mAbs are colored in blue. Only statistically significant features (p-value < 0.001) are shown. The units of the features are summarized in Table S2.

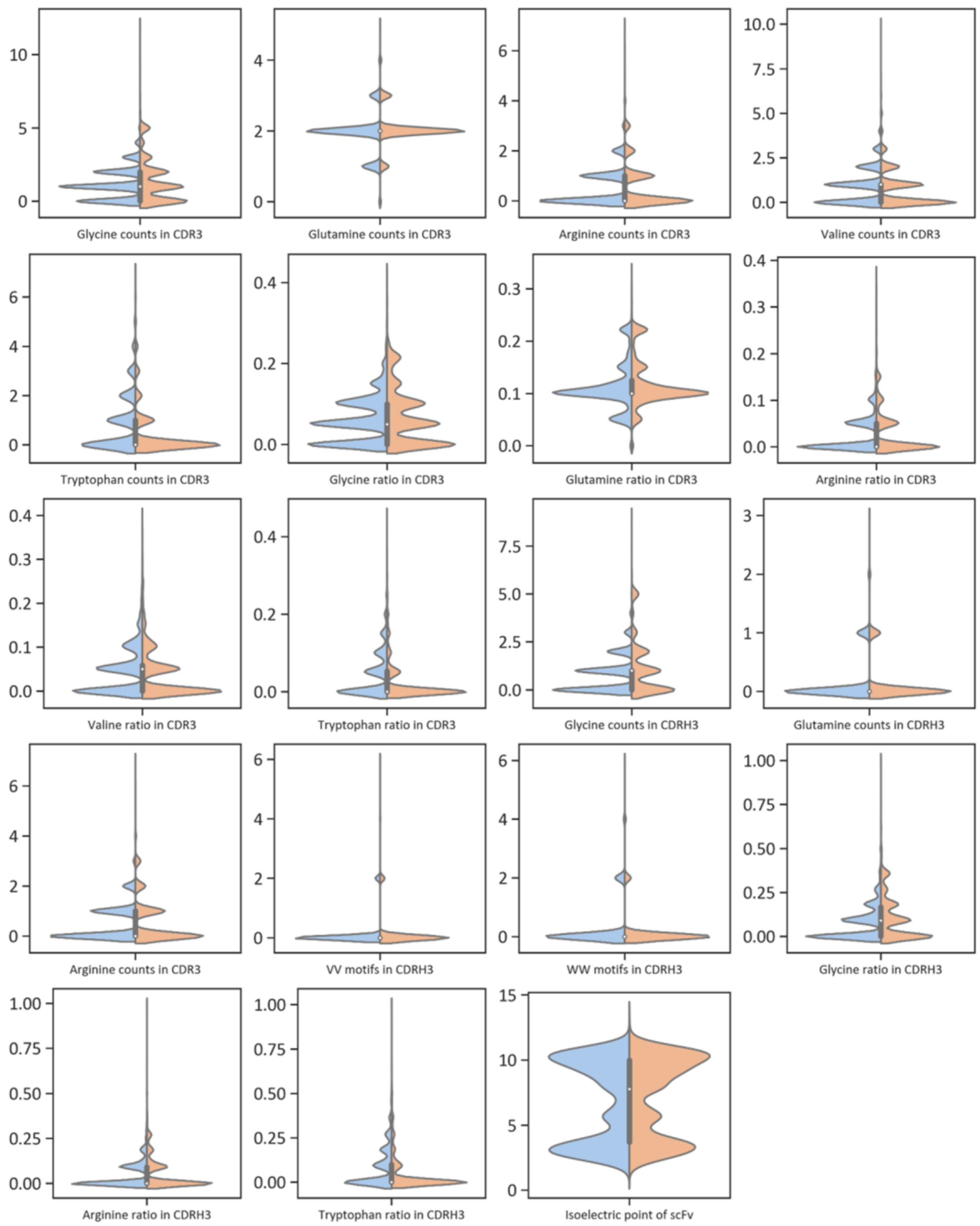

Figure S2. Violin plots for the polyreactive motifs and isoelectric points of scFvs.

Polyreactive mAbs are colored in red, while nonpolyreactive mAbs are colored in blue. Only statistically significant features (p-value < 0.001) are shown. The units of the features are summarized in Table S3.

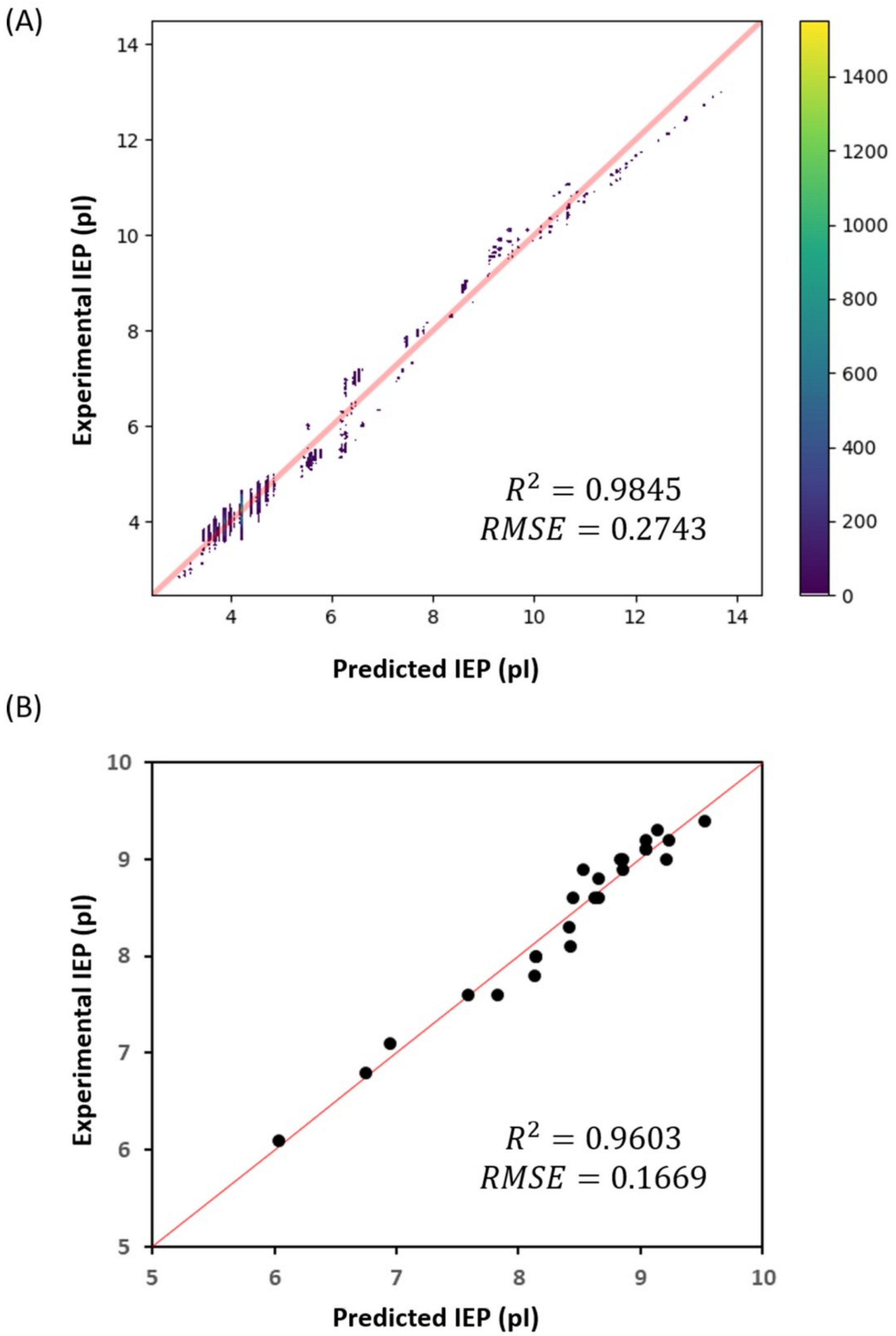

Figure S3. Correlation plots between the experimental IEPs and predicted IEPs.

(A) The correlation plot in 41,943 peptides. (B) The correlation plots in 25 antibodies.

Table S1. Hyperparameter setting for tuning procedure

Method	Tuning parameters	Fixed parameters
GBM	n_estimators = 50, 100, 500, 1000, 1500, 2000, 2500, 3000<br>max_depth = 10, 15<br>max_features = 'auto', 'sqrt', 'log2'<br>learning_rate = 0.01, 0.05	
LGBM	n_estimators = 50, 100, 500, 1000, 1500, 2000, 2500, 3000<br>learning_rate = 0.01, 0.05	class_weight='balanced'
RF	n_estimators = 50, 100, 500, 1000, 1500, 2000, 2500, 3000<br>max_features = 'auto', 'sqrt', 'log2'	class_weight='balanced'
XGB	n_estimators = 50, 100, 500, 1000, 1500, 2000, 2500, 3000<br>max_depth = 10, 15<br>learning_rate = 0.01, 0.05	gamma = 0<br>scale_pos_weight = 10559/8867<br>min_child_weight = 1<br>subsample = 0.5

Table S2. Optimal hyperparameters in this work (continue)

Descriptors	Method	Optimal hyperparameter
F46	GBM	learning_rate = 0.01, max_depth = 10, max_features = 'sqrt', n_estimators = 100
	LGBM	learning_rate = 0.01, n_estimators = 500
	RF	max_features = 'log2', n_estimators = 3000
	XGB	learning_rate = 0.01, max_depth = 10, n_estimators = 500
UniRep	GBM	learning_rate = 0.01, max_depth = 10, max_features = 'auto', n_estimators = 500
	LGBM	learning_rate = 0.01, n_estimators = 500
	RF	max_features = 'sqrt', n_estimators = 1500
	XGB	learning_rate = 0.01, max_depth = 10, n_estimators = 500
TAPE	GBM	learning_rate = 0.01, max_depth = 10, max_features = 'sqrt', n_estimators = 1000
	LGBM	learning_rate = 0.01, n_estimators = 500
	RF	max_features = 'auto', n_estimators = 3000
	XGB	learning_rate = 0.01, max_depth = 10, n_estimators = 500
ESM-1b	GBM	learning_rate = 0.01, max_depth = 10, max_features = 'sqrt', n_estimators = 100
	LGBM	learning_rate = 0.05, n_estimators = 100
	RF	max_features = 'auto', n_estimators = 3000
	XGB	learning_rate = 0.01, max_depth = 10, n_estimators = 500
ESM-1v	GBM	learning_rate = 0.05, max_depth = 15, max_features = 'log2', n_estimators = 3000
	LGBM	learning_rate = 0.01, n_estimators = 500
	RF	max_features = 'sqrt', n_estimators = 500
	XGB	learning_rate = 0.01, max_depth = 10, n_estimators = 50

Table S2. Optimal hyperparameters in this work (continue)

Descriptors	Method	Optimal hyperparameter
F46/UniRep	GBM	learning_rate = 0.01, max_depth = 10, max_features = 'auto', n_estimators = 1000
	LGBM	learning_rate = 0.01, n_estimators = 500
	RF	max_features = 'auto', n_estimators = 1000
	XGB	learning_rate = 0.01, max_depth = 10, n_estimators = 100
F46/TAPE	GBM	learning_rate = 0.05, max_depth = 15, max_features = 'auto', n_estimators = 1500
	LGBM	learning_rate = 0.05, n_estimators = 100
	RF	max_features = 'auto', n_estimators = 2500
	XGB	learning_rate = 0.01, max_depth = 10, n_estimators = 500
F46/ESM-1b	GBM	learning_rate = 0.01, max_depth = 10, max_features = 'auto', n_estimators = 500
	LGBM	learning_rate = 0.05, n_estimators = 50
	RF	max_features = 'auto', n_estimators = 2500
	XGB	learning_rate = 0.01, max_depth = 10, n_estimators = 50
F46/ESM-1v	GBM	learning_rate = 0.01, max_depth = 10, max_features = 'auto', n_estimators = 100
	LGBM	learning_rate = 0.01, n_estimators = 50
	RF	max_features = 'auto', n_estimators = 500
	XGB	learning_rate = 0.01, max_depth = 10, n_estimators = 50

Table S3. The classifying performance of physicochemical properties of scFvs

Class	Feature Name	Unit	AUC	p-value[a]
Net Charge	Net charge of all CDRs	e^-	0.521	***
	Net charge of CDR1	e^-	0.556	***
	Net charge of CDR2	e^-	0.413	***
	Net charge of CDR3	e-	0.521	***
Aggregation	AggScore	unitless	0.506	***
	Zyaggregator	unitless	0.489	***
	Zyaggregator_p	unitless	0.541	***
Solvent-Accessible Surface Area	SASA of all hydrophobic atoms	$Å^2$	0.502	**
	SASA of exposed hydrophobic atoms	$Å^2$	0.512	**
Hydrodynamic properties	Partial specific volume (v_{bar})	mL/g	0.474	*
	Molecular weight (M)	Da	0.470	
	Anhydrous volume sphere radius (R_o)	Å	0.465	
	Anhydrous radius of gyration (R_g)	Å	**0.628**	***
	Maximum dimension (D_{max})	Å	0.598	***
	Axial ratio	unitless	0.584	***
	frictional ratio	unitless	0.600	***
	Translational diffusion coefficients (D_t)	cm^2/s	0.424	***
	Translational hydrodynamic radius (R_{trans})	Å	0.576	***
	Sedimentation coefficients (s)	second	0.426	***
	Rotational diffusion coefficients (D_r)	s^{-1}	0.404	***
	Rotational hydrodynamic radius (R_{rot})	Å	0.596	***
	Tumbling correlation time (tauC)	second	0.596	***
	Asphericity	unitless	0.621	***
CDR Length	Length of all CDRs	count	0.482	*
	Length of CDR1	count	0.393	***
	Length of CDR2	count	0.530	
	Length of CDR3	count	0.545	***

[a] *** $p < 0.001$, ** $p < 0.01$, and * $p < 0.05$

Table S4. The classifying performance of polyreactive motifs and isoelectric points of scFvs

Class	Feature Name	Unit	AUC	p-value[a]
Polyreactive motifs	Glycine (Gly) counts in CDR3	count	0.521	***
	Glutamine (Gln) counts in CDR3	count	0.545	***
	Arginine (Arg) counts in CDR3	count	0.510	***
	Valine (Val) counts in CDR3	count	0.455	***
	Tryptophan (Trp) counts in CDR3	count	0.384	***
	Glycine (Gly) ratio in CDR3	unitless	0.506	***
	Glutamine (Gln) ratio in CDR3	unitless	0.512	***
	Arginine (Arg) ratio in CDR3	unitless	0.507	***
	Valine (Val) ratio in CDR3	unitless	0.447	***
	Tryptophan (Trp) ratio in CDR3	unitless	0.382	***
	Glycine (Gly) counts in CDRH3	count	0.558	***
	Glutamine (Gln) counts in CDRH3	count	0.498	
	Arginine (Arg) counts in CDRH3	count	0.508	***
	Valine (Val) counts in CDRH3	count	0.483	
	Tryptophan (Trp) counts in CDRH3	count	0.383	***
	Two valine motif counts (VV) in CDRH3	count	0.508	***
	Two tryptophan motif counts (WW) in CDRH3	count	0.482	***
	Glycine (Gly) ratio in CDRH3	unitless	0.548	***
	Glutamine (Gln) ratio in CDRH3	unitless	0.498	
	Arginine (Arg) ratio in CDRH3	unitless	0.505	***
	Valine (Val) ratio in CDRH3	unitless	0.477	**
	Tryptophan (Trp) ratio in CDRH3	unitless	0.383	***
Isoelectric points	Isoelectric point of scFv	unitless	0.535	***
	Isoelectric point of CDR	unitless	0.504	*

[a] *** $p < 0.001$, ** $p < 0.01$, and * $p < 0.05$
